# Risk Factors for Severe and Fatal Heat-Related Illness in UK Dogs—A VetCompass Study

**DOI:** 10.3390/vetsci9050231

**Published:** 2022-05-11

**Authors:** Emily J. Hall, Anne J. Carter, Guaduneth Chico, Jude Bradbury, Louise K. Gentle, Dominic Barfield, Dan G. O’Neill

**Affiliations:** 1 Department of Clinical Science and Services, The Royal Veterinary College, Herts AL9 7TA, UK; dbarfield@rvc.ac.uk; 2 School of Animal, Rural and Environmental Sciences, Nottingham Trent University, Notts NG25 0QF, UK; anne.carter@ntu.ac.uk (A.J.C.); guaduneth.chicoleon@ntu.ac.uk (G.C.); louise.gentle@ntu.ac.uk (L.K.G.); 3 Royal College of Veterinary Surgeons, London WC2A 1EN, UK; j.bradbury@rcvs.org.uk; 4 Pathobiology and Population Sciences, The Royal Veterinary College, Herts AL9 7TA, UK; doneill@rvc.ac.uk

**Keywords:** VetCompass, heat-related illness, canine heatstroke, exertional heat-related illness, vehicular heat-related illness, brachycephalic, dogs die in hot cars, dogs die on hot walks

## Abstract

Heat-related illness (HRI) is predicted to increase in dogs due to rising global temperatures. This study evaluated retrospective VetCompass veterinary clinical records to explore geographical variability and ambient conditions associated with HRI events in UK dogs, and report the intrinsic (canine) and extrinsic (location, trigger, ambient weather) risk factors for severe disease and fatal outcome in dogs affected by HRI. Dogs living in London had the greatest odds for developing HRI compared with dogs living in the North West (OR 1.9, 95% CI 1.31–2.74). The median ambient temperature on days of HRI events was 16.9 °C. For dogs with HRI, age, bodyweight and trigger were risk factors associated with severe disease. Age, skull shape and clinical grade of HRI presentation were associated with a fatal outcome. Whilst the majority of HRI events overall were triggered by exertion, the risk of severe disease was greater in situations where dogs could not escape the heat source (vehicular confinement), and the risk of death in HRI cases was greater for those dogs with reduced capacity to thermoregulate (older and brachycephalic dogs). These results highlight the need for better owner awareness of the factors that increase the risk of severe and fatal HRI, as a first stage in protecting canine welfare in the face of rising global temperatures.

## 1. Introduction

As global temperatures continue to climb [1], the urgent development of mitigation strategies to protect both humans [2] and animals [3] from the ill-effects of extreme heat is a One Health priority. Vulnerability assessments are needed to identify populations at greatest risk from heat-related illness (HRI), in order to support the creation of improved adaptation plans and health care approaches [2]. These will allow better responses to extreme heat events, reducing HRI severity and fatality [2]. Domestic dogs play important roles in society, including companionship [4] and fulfilling service roles ranging from military and policing to medical assistance. Climate change mitigation strategies must therefore include consideration of canine-specific risk factors to reduce HRI risk and severity in dogs and maintain the unique positions of dogs in human society [3,5].

Exposure to a hot environment (environmental or vehicular) or physical exertion in any environment [6] can trigger HRI by raising core body temperature beyond homeostatic thermoregulatory capacity [7,8]. Dogs, like humans [9,10], experience grades of HRI, progressing from mild (characterised by lethargy and altered respiration), to moderate (gastrointestinal disorder, episodic collapse and a single seizure) and ultimately severe disease (neurological derangement, bleeding disorders, gastrointestinal haemorrhage, liver and kidney damage) [11]. In a previous study, disease progression from mild/moderate to severe HRI reduced survival from over 90% to 43% [11]. Severe HRI occurs when a dog’s body temperature exceeds a critical temperature threshold of 43 °C; both the degree of temperature elevation and the duration of temperature elevation beyond 43 °C determine the severity of thermal damage [12,13].

Confinement in a hot environment such as a vehicle or building, or intense physical exertion, can overwhelm thermoregulatory capacity [8]. Dogs with impaired thermoregulation from altered anatomy (such as brachycephaly), obesity or underlying health conditions affecting the respiratory or circulatory systems, are at increased risk of developing severe HRI at relatively low ambient temperatures, or with relatively low levels of physical exertion [8,14]. An important aspect of physiological adaptation to coping with hot environments or intense physical exertion is heat acclimatisation [7]. The process of heat acclimatisation can take weeks to develop and requires increasing levels of exertion in a hot environment [7]. In the UK, regular long-term exposure to high ambient temperatures is rare due to the variable climate [15]. Nevertheless, the UK’s geography, including proximity to the Atlantic Ocean, long coastline and topography, results in both seasonal and day to day variation in ambient conditions that can cause sudden exposure to high ambient temperatures without accompanying opportunity for heat acclimatisation [15].

Dogs rely on evaporative cooling through panting once their thermoregulatory set point has been exceeded [16], where ambient temperature, humidity and windspeed all influence the rate of cooling. The wet bulb globe temperature (WBGT) measures ambient conditions, taking air temperature, radiant heat, humidity and windspeed into account to provide an assessment of thermal stress [17]. The upper limit for human survivability under sustained exposure is a WBGT of 35 °C [18], whilst severe mortality and morbidity have occurred during heat waves with a maximum reported WBGT of 28 °C [19]. Equestrian competitions also use WBGT to assess thermal suitability for equine athletes, with a limit of 32.5 °C proposed for competitions involving fit, fully acclimatised horses [20] and a recommendation to implement precautions to reduce heat load when WBGT exceeds 28 °C [21]. No formal guidelines exist for dogs training or working in hot environments. A review of HRI in military working dogs reported that three-quarters of HRI events occurred at a WBGT > 26.7 °C; the lowest recorded WBGT for an event was 20.4 °C [22]. Military working dogs are typically athletically fit, heat-acclimatised dogs with non-extreme conformational phenotypes, suggesting that an average pet dog would likely develop HRI at even lower temperatures. Pet dogs with impaired thermoregulatory ability (such as obese or brachycephalic dogs) would likely be at increased risk of developing HRI at lower temperatures still [8,23,24].

Previous studies have focused on identifying the intrinsic canine risk factors for HRI in dogs [24,25,26] and the most common triggers of HRI in UK dogs [6,27]. However, these studies have not explored combined effects from intrinsic and extrinsic factors such as HRI trigger, ambient condition and geographical location, on HRI severity or fatality. Severe HRI can have a negative impact on canine welfare on both an immediate (pain, distress and separation from their familiar home environment) and long term (ongoing organ dysfunction, reduced ability to exercise) basis [28], and over 50% of UK dogs presenting to primary care veterinary practices with severe HRI symptoms died as a result of the condition [11]. Better understanding of the combined risk factors for severe and fatal HRI is therefore needed to support the formulation of heat mitigation strategies for dogs. This will facilitate rapid recognition of high-risk patients presenting for veterinary care, and support more targeted owner education campaigns to improve canine welfare.

This study aims to (1) present the geographical variability of HRI incidence in UK dogs; (2) report the ambient conditions when canine HRI has occurred; and (3) explore the intrinsic (canine) and extrinsic (location, trigger and ambient weather) risk factors for severe disease and fatal outcome in dogs affected by HRI.

## 2. Materials and Methods

### 2.1. Data Collection and Management

This study continues the work previously reported by Hall et al. and used the same dataset described in those studies [6,11,24]. The VetCompass Programme shares deidentified electronic patient records (EPRs) from affiliated UK veterinary practices to generate an online research database including over 9 million dogs for large-scale epidemiological studies. This study included clinical data from 905,543 dogs under veterinary care at primary care practices during 2016, as previously defined [24]. Candidate cases for HRI were identified by searching EPRs for the following terms: heat stroke~3, heatst*, hyperthermi*, overheat*, over heated~2, heat exhaustion~2, hot car~2, collapse* + heat, cooling, high ambient temp*. Candidate cases were manually reviewed by two authors (authors 1 and 2) to identify all dogs meeting the case definition of HRI and presented for management of HRI between 1 January 2016 and 31 December 2018 (defined in Hall et al. [24]). Only HRI events occurring between 1 January 2016 and 31 December 2018 were included in the analysis, as HRI events that occurred prior to 1 January 2016 resulted in a survival outcome to at least 2016 by definition [6].

Confirmed HRI events underwent further data extraction including: breed, age at event, mean lifetime adult body weight, skull shape, HRI trigger [6], date of the HRI event, HRI event outcome (survived to discharge or died), and partial postcode area. Cases with an outcome of ‘died’ were further classified by mechanism of death: euthanasia, unassisted or unknown. Where the dog’s clinical history reported over 24 h delay between the event that triggered the HRI and presentation to the veterinary practice, the date of event was either accurately determined (e.g., “the dog overheated 2 days ago and is still unwell”), or estimated (e.g., the dog was treated for heatstroke whilst on holiday last week). The dog’s breed was used to determine skull type: mesocephalic, dolichocephalic, brachycephalic, brachycephalic cross or unknown (see Hall et al. [24] for definitions). All events were retrospectively graded using the VetCompass Clinical Grading Tool for HRI in Dogs [11].

### 2.2. Geographical Distribution of Cases

The approximate location (postcode area) of each HRI and non-HRI case was determined by the initial alphabetical portion of the partial postcode recorded in the dog’s EPR. The HRI incidence risk for the UK as a whole and for each postcode area was calculated using the overall demographic data of 905,543 dogs under veterinary care during 2016 [24], and HRI cases that occurred between 1 January 2016 and 31 December 2016. The 95% confidence interval (CI) for the incidence risk was calculated using EpiTools [29]. The distribution maps of the HRI incidence risk per postal town, and HRI case fatalities (including all mechanisms of death) per postal town were created using ArcGIS 10.8. HRI cases, HRI case incidence and distribution of HRI fatalities data were linked to the UK postcode areas vector data to display the geographical distribution.

The postcode area extracted from each HRI case’s EPR was used to determine the Nomenclature of Territorial Units for Statistics (NUTS) level 1 United Kingdom region code [30]. Where no postcode area was recorded in the dog’s EPR, a classification of “unrecorded” was used accounting for 74,082/905,543 (8.18%) cases. The regional HRI incidence was calculated using the overall demographic data and HRI cases that occurred during 2016 (as above). A univariable binary logistic regression model was used to explore the regional risk for HRI in UK dogs; the region with the greatest denominator population of dogs was used as the comparator.

Using all HRI events from 1 January 2016 to 31 December 2018, the number of HRI events caused by each of the four main triggers (exertion, environment, vehicular and building confinement) was identified for each region, excluding HRI events with no known trigger. This cumulatively accounted for 559/592 (94.4%) of events with a known trigger. At a univariable level, the odds ratio for each trigger by region versus the rest of the UK was calculated using MedCalc [31], and events from dogs with an unknown region were excluded from this analysis.

### 2.3. Ambient Conditions

The date of the HRI event and partial postcode locations were used to determine an estimate of the ambient temperature for each individual HRI event using the Met Office’s MIDAS Open: UK Land Surface Stations Data (1853–current) [32]. Events with an estimated date of event or missing partial postcode were excluded from this analysis. The maximum daily WBGT was extracted for the day of each HRI event using the date of exposure from the dog’s EPR, and the nearest available weather station based on the partial postcode.

Summary statistics were calculated using HRI events from 2016 to 2018 to explore the variation in WBGT for the different HRI triggers. Variation in WBGT distributions between outdoor triggers (environmental, exertional and vehicular) of HRI were compared using a Kruskal–Wallis test with Dunn’s pairwise post hoc analysis. Statistical significance was indicated by *p* < 0.05 (adjusted using the Bonferroni correction).

### 2.4. Risk Factor Analysis

Demographic and clinical data were exported to Microsoft Excel (v16, Redmond, WA, USA) for cleaning and descriptive analysis. Statistical and risk factor analysis used SPSS v28 (IMB Inc., Armonk, NY, USA). Multivariable binary logistic regression modelling was used to identify risk factors for severe HRI and fatal HRI, separately.

To identify risk factors for severe HRI, the denominator population included only HRI cases presented between 2016 and 2018 and categorised using the VetCompass Clinical Grading Tool in the analysis, with events classified as “severe” as the outcome of interest. Risk factors considered in the model included categorical canine variables defined in Table 1 [24], UK region (including the 12 NUTS UK regions alongside an “unrecorded” category for dogs with no postcode recorded in the EPR), HRI trigger (as defined by Hall et al. [6]), and the continuous variable WBGT (°C).

To identify risk factors for fatal HRI, all HRI cases presented between 2016 and 2018 were included in the denominator population with cases recorded with an outcome of “death” from any mechanisms (unassisted, euthanasia and unknown) as the outcome of interest. Risk factors considered in the modelling included categorical canine demographic variables defined in Table 1 [24], UK region (including the 12 NUTS UK regions alongside an “unrecorded” category for dogs with no postcode recorded in the EPR), HRI trigger (as defined by Hall et al. [6]), VetCompass Clinical Grading Tool classification (mild, moderate, severe or ungraded [11]) and the continuous variable WBGT (°C).

Variables correlated with breed (skull shape and bodyweight) were explored separately, pairwise interactions were explored and retained where biologically meaningful. Risk factors with liberal associations in univariable modelling (*p* < 0.2) were selected for multivariable evaluation; model development used a manual backwards stepwise elimination. The area under the receiver operating characteristic (ROC) curve [33] and R^2^ were used to evaluate the explanatory ability of the model alongside consideration of the underpinning biological plausibility of the model specification. The Akaike information criterion (AIC) was used to compare and select the final models, with a lower AIC indicating model superiority.

## 3. Results

### 3.1. Geographical Distribution of HRI Events

**Figure 1:**
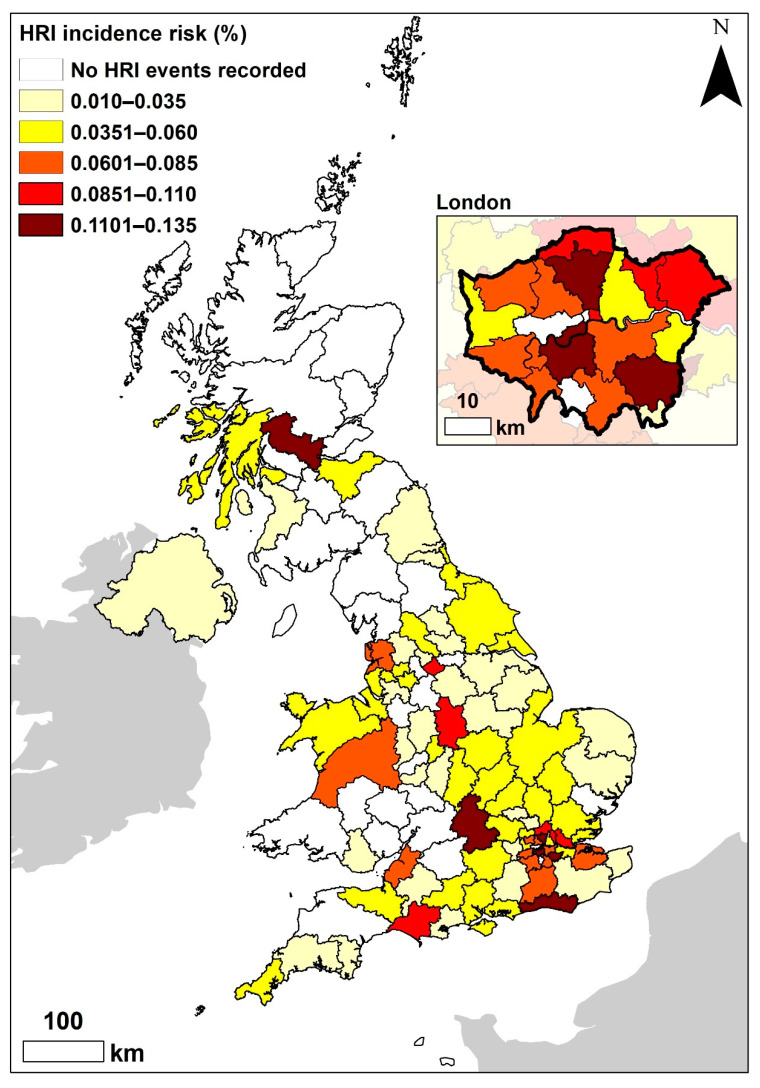
Incidence risk (%) of heat-related illness (HRI) in dogs under primary veterinary care during 2016 by UK postcode region.

**Figure 2:**
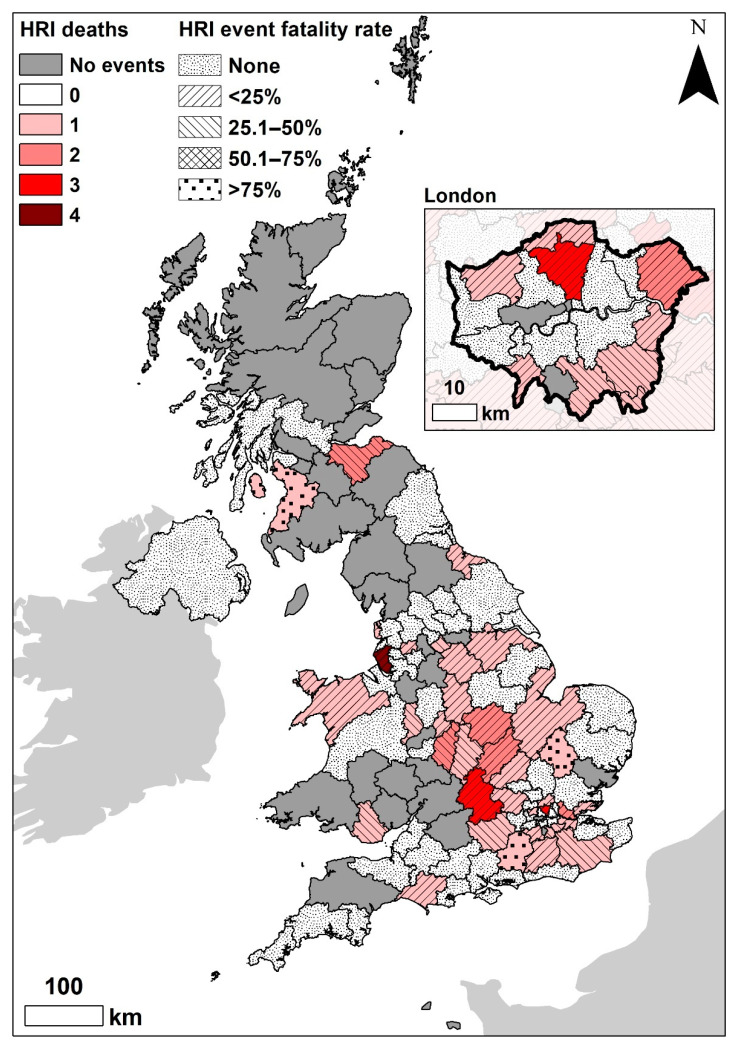
Distribution of heat-related illness (HRI) fatalities in dogs under primary veterinary care during 2016 by UK postcode region for 2016. The HRI event fatality rate for each region is overlaid.

There were 395 HRI events recorded in 2016, involving 390 individual dogs (Table 2). The affected dogs were distributed across 88/121 UK postcode areas, across all four countries of the UK, resulting in the overall estimated 2016 HRI incidence risk of 0.04% (95% CI 0.04–0.05) [24]. The postcode areas with the highest HRI incidence risks were North London (0.14%, 95% CI 0.07–0.24%), Brighton (0.13%, 95% CI 0.02–0.75%), Bromley (0.12%, 95% CI 0.04–0.34%) and Oxford (0.12%, 95% CI 0.07–0.19%) (Figure 1). The overall 2016 HRI event fatality rate has been reported previously 14.18% (95% CI 11.08–17.96%) [24]. The postcode areas with the highest number of deaths were Liverpool (four), North London (three) and Oxford (three) (Figure 2). The regions with the highest HRI event fatality rates were Cambridge, Guildford and Kilmarnock (Figure 2); however, these rates represent a fatal outcome for the only (single) HRI event recorded in each postcode area.

At a regional level, compared to dogs living in the North West of England, dogs living in London had 1.9 times the odds of developing HRI during 2016. The 2016 regional incidence of HRI in dogs was highest in London (0.083%) and lowest in Northern Ireland (0.012%) (Table 3).

The 856 HRI events that occurred between 2016 and 2018 included 264 (30.84%) without a trigger recorded in the EPR. The events with known triggers are shown in Table 4, presented by NUTS UK region. The percentage of events triggered by exertion was highest in the South East with 2.15 times the odds (95% CI 1.44–4.02) compared to the rest of the UK. The percentage of events triggered by environmental heat was highest in the North East with 3.05 times the odds (95% CI 1.23–8.29) compared to the rest of the UK. The percentage of events triggered by vehicular confinement was highest in Wales with 4.17 times the odds (95% CI 1.11–15.69), and lowest in London with 0.13 times the odds (95% CI 0.02–0.99), compared to the rest of the UK. The percentage of events triggered by confinement in hot buildings was highest in London with 3.36 times the odds (95% CI 1.27–8.92) compared to the rest of the UK.

### 3.2. Ambient Temperature

**Figure 3:**
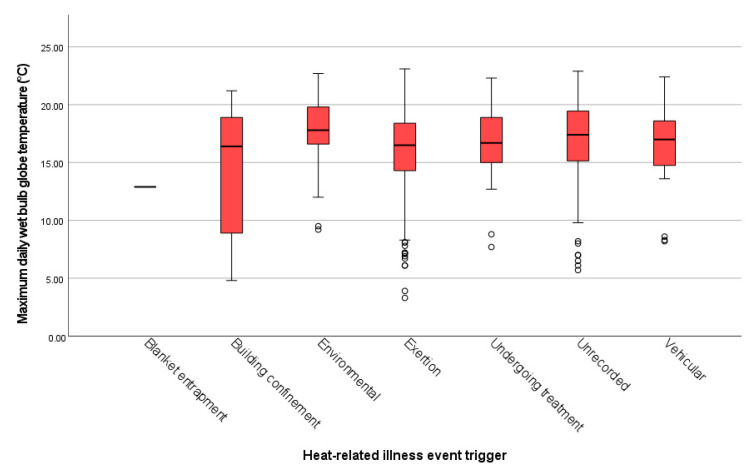
Median (± interquartile range and outlier values indicated by circles) of maximum daily wet bulb globe temperature (°C) for heat-related illness events in UK dogs between 2016 and 2018 by event trigger type.

The maximum daily WBGT recorded on days of HRI events ranged from 3.3 to 23.1 °C (median 16.9 °C, IQR 14.8–18.9 °C). The variation in WBGT by HRI event trigger is shown in Figure 3. The distribution of WBGT was significantly different across outdoor triggers of HRI (environmental, exertional, and vehicular) (H = 16.23, *p* < 0.001). Dunn’s pairwise post hoc analysis (adjusted using the Bonferroni correction) indicated that the WBGT recorded for environmental HRI events (17.8 °C) was significantly higher than the WBGT recorded for exertional (16.5 °C) HRI events (*p* < 0.001).

### 3.3. Risk Factor Analysis for Severe HRI

At a univariable level, for dogs with HRI, the variables trigger (*p* = 0.008), age (*p* = 0.040), bodyweight (*p* = 0.003), bodyweight relative to breed/sex mean (*p* < 0.001), NUTS region (*p* = 0.193) and the interaction between trigger and WBGT (*p* = 0.009) were liberally associated with severe HRI, whereas skull shape (*p* = 0.811), breed type (*p* = 0.837), sex/neuter (*p* = 0.533) and WBGT (*p* = 0.357) were not. The final multivariable model retained three variables: trigger, age and bodyweight, with good discrimination (R^2^ = 0.137, area under ROC curve: 0.719). Dogs with HRI events triggered by vehicular confinement (OR 3.03, 95% CI 1.28–7.15) and dogs with no trigger recorded in the event history (OR 1.69, 95% CI 1.05–2.72) had increased odds of severe HRI compared to dogs with exertional HRI (Table 5). Compared to dogs aged under 2 years, dogs aged 12 years or over had the greatest odds for severe HRI (OR 5.89, 95% CI 2.51–13.82), and dogs aged 2 to <8 years old had increased odds for severe HRI. Compared to dogs weighing under 10 kg, dogs with no bodyweight recorded in the EPR had the greatest odds for severe HRI (OR 6.46, 95% CI 2.84–14.71) and dogs weighing 10 to <20 kg had increased odds for severe HRI (OR 2.71, 95% CI 1.17–6.28).

### 3.4. Risk Factor Analysis for Fatal HRI

In the univariable analysis, for dogs with fatal HRI, the variables VetCompass Clinical Grade (*p* < 0.001), HRI trigger (*p* = 0.001), skull shape (*p* = 0.152), age (*p* < 0.001), bodyweight (*p* = 0.001), bodyweight relative to breed/sex mean (*p* = 0.001), WBGT (*p* = 0.067) and the interaction between trigger and WBGT (*p* < 0.001) were liberally associated with fatal HRI, whereas the variables breed (*p* = 0.925), NUTS region (*p* = 0.385), and sex/neuter (*p* = 0.266) were not. The final multivariable model retained three variables: VetCompass Clinical Grade, skull shape and age, and showed good discrimination (R^2^ = 0.447, area under ROC curve: 0.895). Compared to dogs aged under 2 years, dogs aged 12 years or over had the greatest odds of fatal HRI (OR 8.87, 95% CI 2.87–27.41) (Table 6). Compared to dogs with a mesocephalic skull shape, dogs with a brachycephalic skull shape had greater odds (OR 3.01, 95% CI 1.60–5.67) of fatal HRI. When compared to HRI events graded as mild, dogs graded as moderate (OR 2.70, 95% CI 1.11–6.55), severe (OR 64.92, 95% CI 27.12–155.43) and unclassified (OR 8.05, 95% CI 2.73–23.74) had greater odds of fatal HRI.

## 4. Discussion

Although the incidence of HRI in UK dogs is relatively low, it is a potentially fatal yet preventable condition. Identifying factors associated with severe and fatal outcomes in dogs presented for veterinary treatment of HRI is an important step in planning mitigation strategies to protect all domestic dogs in the face of rising global temperatures. Dogs that developed HRI following confinement in a hot vehicle had three times the odds of developing severe HRI compared to dogs that developed HRI following exercise. Increasing age was associated with both severe and fatal HRI, with dogs aged 12 years or older having the greatest odds of both when compared to dogs aged under 2 years old. Skull shape was associated with increased odds of fatal HRI, but not severe HRI. Brachycephalic dogs with HRI had three times the odds of a fatal outcome compared to mesocephalic dogs with HRI.

Previous studies have identified risk factors associated with developing HRI in dogs, including skull shape, bodyweight, age, breed and being overweight [24,25,26]. Whilst any dog can develop HRI if exposed to a high enough ambient temperature for long enough, brachycephalic dogs [24,25,27], overweight dogs [24,25,34], heavier dogs [24] and dogs aged 2–8 and over 12 years [24] have greater odds of developing HRI. However, our previous study [24] explored risk factors for the entire spectrum of severity of HRI presented for veterinary care and did not distinguish between differing risk factors across different severity grades of HRI [6,24,27], with only 14% of dogs developing severe disease [11,27] and a survival rate over 95% for dogs with mild/moderate disease [11]. This present study aimed to identify risk factors for HRI progression to severe or fatal disease within those dogs that were recorded with HRI; this information could facilitate more effective triage of affected dogs.

In the current study, trigger, age and bodyweight were found to be risk factors. The lack of inclusion of skull shape as a criterion for severe HRI potentially highlights the additional conformational challenges faced by brachycephalic breeds in relation to HRI as brachycephalic breeds had greater odds of death. When experiencing mild or moderate HRI, brachycephalic dogs with airway restrictions, such as those associated with brachycephalic obstructive airway syndrome (BOAS), can develop respiratory arrest [35] and cardiac arrest (death) before severe HRI is reached. It is therefore vital that early signs of mild HRI are recognised and acted upon as dogs with respiratory disorders (including BOAS) are more likely to escalate into fatal difficulty before advanced signs of HRI occur. Dogs aged over 12 years had both increased odds of severe and fatal HRI, which is likely due to the diminished capacity of older dogs to thermoregulate [36]. However, age (measured in years) is a relatively poor measure of life stage in dogs as bodyweight is known to influence longevity [37]. Whilst younger dogs have reduced odds of severe and fatal HRI, they are still capable of developing the condition and this risk should not be ignored.

Dogs without a recorded trigger and a recorded bodyweight had greater odds of severe HRI. Whilst these results do not appear to provide meaningful clinical information to aid decision making, they may reflect a rapid veterinary response in prioritising treating and cooling severe HRI cases over recording details related to the incident. The increased odds of severe HRI for dogs without a recorded trigger in their clinical history also supports the need for better owner education regarding the causes of HRI, and the recognition of clinical signs that indicate the onset of mild to moderate HRI [11]. Without an awareness that their dog could be affected by HRI following exercise, many owners may not recognise the condition or grasp the urgency of the situation, which could result in failure to seek veterinary care until the dog had progressed to severe HRI. As dogs presented for veterinary treatment with severe HRI had almost 65 times the odds of death, improving the ability of owners to recognise the early clinical signs of mild to moderate disease should be considered an urgent priority for mitigating the risk of HRI.

This study explored the geographical distribution of HRI events in UK dogs, and identified that dogs living in London had almost twice the odds of HRI during 2016 than dogs living in the North West; the 2016 incidence of HRI in London (0.08%) was twice the national HRI incidence (0.04%). This could be due to the urban ‘heat island phenomenon’ [38]; the ambient temperature in cities such as London can be around 5 °C hotter than the surrounding countryside [39], and the housing in cities frequently includes higher proportions of apartments and terraced houses than rural locations. Both flats and terraced housing are generally located within the warmest parts of cities [40] and are associated with an increased risk of overheating [38]. Whilst it does not explain all the additional HRI events in London, a significantly greater percentage of HRI cases in London were triggered by confinement in a hot building (7.61%) compared to the rest of the UK (2.39%).

Conversely, the percentage of HRI events in London triggered by vehicle confinement (1.09%) was significantly lower than the rest of the UK (7.61%). Reports to authorities regarding dogs being left in hot vehicles are more likely in high socioeconomic regions [41]. However, it is unclear if this is because people living in higher socioeconomic regions are more likely to report a dog in a hot vehicle or because people living in higher socioeconomic regions are more likely to own a car and drive their dog to their outdoor exercise [41]. The reduced incidence of vehicular HRI in London is potentially explained by the difference in transport preferences, with just 58% of London households owning a vehicle compared to 83% of households in the remainder of England [42]. However, decreased vehicle ownership is unlikely to be the only factor impacting the reduced incidence of vehicular HRI in London so further research could be considered to explore other influential factors such as improved public awareness of the risk, or concern over dog thefts reducing owner willingness to leave dogs in cars. An earlier study reported that UK vehicular HRI events occurred from March to September [6] when ambient temperatures are typically warmer. In this study, vehicular HRI events also occurred on days when the maximum recorded WBGT was <10 °C. Internal vehicle temperatures are predominantly influenced by solar radiation [43], and UK vehicles have been reported to exceed the upper limit of the canine thermoneutral zone (35 °C) between April and September and exceed 50 °C between May and August [44].

Bruchim et al. [25] reported that only 4 of 54 dogs with severe HRI presented to a veterinary hospital in Israel had been exposed to ambient temperatures below 22.1 °C, whilst 46/54 had been exposed to an ambient discomfort index (the mean of the WBGT and dry bulb globe temperature) of at least 24.1 °C. In comparison, the highest maximum daily WBGT recorded in the present study was 23.1 °C, suggesting that UK dogs developed HRI at relatively low ambient temperatures compared to dogs from a country where heat acclimatisation is possible. The median maximum daily WBGT when UK dogs presented with exertional HRI was 16.5 °C in the current study, which is much lower than the temperatures typically associated with exertional HRI in US military working dogs (>26.7 °C) [22]. The lowest maximum daily WBGT recorded in the present study (3.3 °C) was for a HRI event triggered by exertion during the winter. Dogs in the UK have been recorded with post-exercise temperatures of 42.5 °C in ambient temperatures of −5.5–11.7 °C [45], highlighting the potential for dogs to develop HRI following exercise even in relatively or extremely low temperatures. A lack of acclimatisation opportunities, due to the variable UK climate, may contribute to the high proportion of HRI events triggered by exercise in UK dogs compared to other regions. Poor physical fitness and exercise restriction negatively affect thermoregulation effectiveness and reduce exercise endurance [46,47]; therefore, maintaining canine activity throughout the cooler winter months could help to better prepare dogs for warmer summer temperatures.

The present study found that dogs with vehicular HRI had three times the odds for severe HRI compared to dogs with exertional HRI. This is likely due to several important differences between exertional and vehicular HRI, primarily that dogs confined to hot vehicles have no opportunity to escape the heat and can be exposed to more extreme temperatures [44]. Dogs that develop exertional HRI are also more likely to do so in the presence of their owner who can take steps to limit progression of the condition such as ceasing the exercise, seeking shade, and initiating active cooling with water. Dogs left in a hot vehicle may not be found until the HRI has progressed to severe disease or even death. These findings reinforce the ongoing need for owner education campaigns warning that “Dogs Die in Hot Cars” [48], especially given the rise in calls to the RSPCA in both the UK and Australia reporting dogs confined in hot vehicles in recent years [49,50].

Educational campaigns regarding the risk of exertional HRI in dogs requires a different style of messaging to the earlier campaign on “Dogs Die in Hot Cars” [48]. Messaging about exercise must be more nuanced because unintended consequences from overall reduced activity could inadvertently increase canine HRI risk due to obesity and lack of fitness [8,24,46]. The “Dogs Die in Hot Cars” campaign is a call to action: because a dog left unattended in a hot car is at immediate and high risk of developing severe or fatal HRI, owners are urged to never take this risk and the public are encouraged to immediately alert the authorities [48]. In comparison, we show in the current study that exertional HRI is less likely to lead to a severe grade of HRI, especially if the dog is under direct supervision where the owner can take remedial action. Recognition of early clinical signs of HRI disease can allow owners to take immediate action to prevent the dog’s condition worsening (ceasing activity, seeking shade, cooling), and over 95% of dogs presented for veterinary treatment with mild HRI survive [11]. Educational messaging regarding exertional HRI should therefore focus on encouraging owners to learn to recognise early signs of HRI and how to respond to those signs. Owners should also be advised on strategies to reduce the risk of exertional HRI by avoiding activity during the hottest times of the day, adjusting the duration and intensity of canine activity during warmer weather, and recognising that sudden hot spells—particularly in early spring/summer—are especially dangerous.

It must be noted that the present study relied on weather station data for ambient temperatures, using an approximate location based on the address recorded in the dog’s EPR. These data are therefore an estimation of the local environmental (shaded) conditions and are unlikely to fully represent the true conditions actually experienced by the dogs [40]. A recent US study compared ambient temperature data from thermometers mounted on dogs’ collars to neighbourhood and local weather station temperature records [51]. That study reported that the dog-mounted thermometers generally recorded lower ambient temperatures than the local weather station reported during the day for indoor housed dogs, but dogs housed outdoors in urban locations recorded higher local ambient temperatures than either the neighbourhood or regional weather stations. This difference is likely due to the urban heat island phenomenon [40], as the substrate of the outdoor environment (e.g., tarmac [52], grass or artificial grass [53]), presence or absence of shade, and barriers to airflow can all create microclimates that could result in the dog being exposed to far greater heat stress than suggested by the local weather station data.

Moon et al. [51] also found that the temperatures recorded from the dog-mounted thermometers in urban environments were significantly higher than the concurrent weather station temperatures at night. This difference was attributed to reduced airflow within houses, resulting in hotter indoor temperatures than outdoor overnight. Heat stress experienced both during the day and at night contribute to heat-related mortality in humans [54], with heat-sensitive sub-populations (including the elderly and hospital patients) most likely to be located in areas with the highest night-time temperatures [40]. We echo the suggestion by Moon et al. [51] that traditional measures of local temperature, such as heat index data from local weather stations, are likely to underestimate the risk of heat exposure for dogs. Further research aimed at predicting heat risk for dogs in different locations and housing types is needed.

HRI linked to vehicular or building confinement is not only of concern for dogs but is also an issue for more vulnerable people who are unable to easily remove themselves from the situation, including children and babies left in hot cars [55] and elderly individuals confined in buildings [56]. During 2017–2018, almost 3000 people were hospitalised due to severe HRI in the UK [57], giving an approximate population incidence of 0.005%. Our previous study reported an annual HRI (including all grades of HRI) incidence of 0.14% for UK dogs in 2016 [24]. Applying the VetCompass Clinical Grading Tool to just the 2016 canine HRI events provides an estimated annual population severe HRI incidence risk of 0.006% (95% CI 0.005 to 0.008), suggesting that the incidence of severe HRI may be similar between dogs and humans. The dog, therefore, potentially provides a sentinel population for what will become a growing problem in people as global temperatures rise [58].

Improved awareness regarding the risk of HRI from building confinement is needed, particularly within, but not limited to, older dogs and breeds (e.g., brachycephalic) less able to effectively thermoregulate. The findings of the current study highlight some important areas for further research. Due to the relatively small numbers of HRI cases triggered by confinement in buildings and in this present study, limited conclusions can be drawn regarding the differences in geographical distribution of HRI events triggered by building confinement. However, ensuring that city housing is designed or can be adapted to limit heat stress is an important strategy for protecting human health in the face of rising global temperatures [40]. These same mitigations also appear to be important for protecting canine welfare. As the housing with the greatest heat stress risk is frequently associated with the more vulnerable members of society (e.g., aging and hospitalised populations) [40], consideration also needs to be given to assistance dogs (e.g., service dogs for those with vision or hearing impairment) supporting these communities. Several breeds commonly used as service dogs (Labrador Retriever, Golden Retriever, English Springer Spaniel) have been found to have increased odds of exertional HRI compared to non-designer crossbred dogs [6]. Caregivers of these dogs may require more specialised guidance on how to recognise the early stages of HRI as visual impairments may restrict capacity to recognise some of these signs.

The limitations of this study are largely related to the use of historical veterinary patient records as the data extracted from the VetCompass database were not generated for the primary purpose of research [6,11,24]. This has resulted in missing or incomplete data, and some variables (such as location and date of the HRI event) that are imprecise. Diagnosis of HRI is typically based on patient history and presenting clinical signs, and is therefore reliant upon the clinician’s subjective opinion [27], which could have resulted in misidentification for some of the dogs in this study. As previously noted, some owners may not present their animal for veterinary treatment; thus, the true number of HRI events is likely to be higher than reported here [6,24]. The VetCompass Clinical Grading Tool was proposed based on analysis that used the same dataset presented in this present study [11], specifically using a fatal outcome to identify the clinical signs associated with a severe outcome. There is therefore an element of circular logic presented within this analysis, meaning further evaluation of this Clinical Grading Tool using a novel dataset is recommended prior to clinical application. Despite the overall study population of 905,543 dogs, the number of HRI events included in the study is relatively small, which reduces the confidence in some of the results. In particular, the results from the analysis of geographical variation in triggers and ambient temperature of HRI events lack precision due to the uncertainty of the data; they should be considered hypothesis-generating at this stage and require further study before their significance can be fully determined. Finally, the relatively low R^2^ value of both multivariable models suggests that an important variable is missing from the overall model. The duration and degree of body temperature elevation is the single most important factor in determining HRI severity and fatality, which is impossible to measure retrospectively. Therefore, the aim of this paper was to identify other factors that increase the likelihood of that elevation occurring whilst recognising that all dogs are potentially at risk of developing HRI; thus, a truly predictive model is not currently possible to achieve.

## 5. Conclusions

As global temperatures continue to increase, both the incidence of HRI and the frequency of severe disease and fatal outcomes for HRI events are predicted to rise. This study explored combined intrinsic (canine) and extrinsic (geographical location, ambient conditions, and trigger) risk factors for severe and fatal outcomes in dogs presenting to veterinary clinics with HRI. Vehicular confinement and aging (>12 years) were associated with greater odds of severe HRI, whilst aging (over 10 years), clinical grade and brachycephalic skull shape had greater odds of fatal HRI. London saw higher rates of HRI related to building confinement, Wales the highest in relation to vehicular confinement, and the southeast saw higher levels of exertional HRI. Whilst overall the most common trigger of HRI in dogs was exercise, the findings of this study highlight the increased risk of severe and fatal HRI associated with situations where dogs cannot escape the heat source (vehicular confinement) or have reduced capacity to thermoregulate (older dogs and brachycephalic breeds). Dogs living in London had the greatest odds for HRI, and a significantly greater proportion of HRI events were triggered by confinement in hot buildings than the rest of the UK. The relatively low ambient temperatures associated with HRI events in UK dogs—median WBGT of 16.9 °C on days of HRI events—highlight the importance of educating dog owners to recognise the early signs of HRI and be aware that HRI can occur even in relatively mild weather conditions. This improved understanding of the risk factors associated with severe and fatal HRI highlights the need for more targeted educational campaigns to improve public awareness of the different triggers, canine risk factors and clinical signs of HRI in pet dogs.

## Figures and Tables

**Table 1:** Canine risk factors assessed for association with severe/fatal outcomes in dogs affected by heat-related illness (HRI).

Potential Risk Factor for HRI	Variable Definition
Breed type	Categorical variable including all named breed types (including both Kennel Club recognised purebred and non-Kennel Club recognised purebred) and designer hybrid types with contrived names (e.g., Cockapoo, Labradoodle, Lurcher) with ≥5 HRI cases and/or ≥5000 dogs in the overall study population. All remaining dogs were assigned to grouped categories of “other purebred”, “other designer cross” or “non-designer crossbred”.
Skull shape	Purebred dogs were categorised by skull shape into three groups, “brachycephalic”, “mesocephalic” and “dolichocephalic”. Crossbred dogs including a brachycephalic breed were classified as “brachycephalic cross” and all other dogs listed as crossbred or unrecorded breed were classified as “skull shape unrecorded”.
Adult bodyweight	Adult bodyweight was defined as the mean of all bodyweight (kg) values recorded for each dog after reaching 18 months old. Bodyweight (kg) was then categorised into seven groups (<10, 10 to <20, 20 to <30, 30 to <40, 40 to <50, ≥50), dogs under 18 months or with no recorded adult bodyweight were classified as “unrecorded”.
Bodyweight relative to breed/sex mean	Categorical variable grouping dogs with a mean adult bodyweight “equal or above” or “below” the typical (mean) adult bodyweight for their breed and sex (calculated using the overall VetCompass study population). An “unrecorded” variable included all dogs with no adult bodyweight or labelled as crossbred.
Age	The age of the dog at the HRI event. Age (years) was categorised into eight groups (<2, 2 to <4, 4 to <6, 6 to <8, 8 to <10, 10 to <12, ≥12) with “unrecorded” for any dogs with no date of birth recorded in the EPR.

**Table 2:** The number of heat-related illness (HRI) cases and events presenting to primary care veterinary practices in the UK with and without triggers during 2016 and during 2016–2018.

Period	HRI Cases (*n*)	HRI Events (*n*)	HRI Events with No Recorded Trigger (*n*)	HRI Events with a Recorded Trigger (*n*)
2016 only	390	395	122	273
2016–2018	839	856	264	592

**Table 3:** Descriptive and univariable logistic regression results for NUTS UK Region associated with heat-related illness in dogs under primary veterinary care in the VetCompass program in the UK during 2016.

NUTS UK Region	Population (*n =* 905,543)	Case Number (*n* = 390)	2016 HRI Incidence (%)	Odds Ratio	95% CI	*p*-Value
North West	108,005	47	0.044	Reference		
London	87,343	72	0.082	1.90	1.31–2.74	<0.001
South East	100,034	54	0.054	1.24	0.84–1.83	0.279
East Midlands	75,984	33	0.043	1.00	0.64–1.56	0.994
East of England	99,576	43	0.043	0.99	0.66–1.50	0.973
South West	56,116	22	0.039	0.90	0.54–1.50	0.686
West Midlands	74,370	26	0.035	0.80	0.50–1.30	0.371
Yorkshire	101,014	32	0.032	0.73	0.46–1.14	0.167
North East	49,576	14	0.028	0.65	0.36–1.18	0.156
Scotland	36,162	9	0.025	0.57	0.28–1.17	0.125
Wales	32,978	7	0.021	0.49	0.22–1.08	0.076
Northern Ireland	8718	1	0.012	0.26	0.04–1.91	0.187
Unrecorded	74,082	30	0.041	0.93	0.59–1.47	0.758

**Table 4:** The number of heat-related illness (HRI) events triggered by exertion, environmental heat, vehicular confinement and building confinement by NUTS UK region for dogs presenting with HRI to primary care veterinary practices during 2016–2018.

Region	HRI Events with a Recorded Trigger (*n* = 592)	Inciting Heat-Related Illness Event Trigger			
		Exertion No. (%)	Environmental No. (%)	Vehicular No. (%)	Building No. (%)
Whole of UK	592	420 (70.95)	84 (14.19)	37 (6.25)	18 (3.04)
London	92	63 (68.48)	13 (14.13)	1 (1.09) *	7 (7.61) *
East of England	77	52 (67.53)	14 (18.18)	8 (10.39)	1 (1.30)
South East	75	62 (82.67) *	6 (8.00)	5 (6.67)	2 (2.67)
North West	67	47 (70.15)	9 (13.43)	5 (7.46)	1 (1.49)
Yorkshire and The Humber	65	47 (72.31)	10 (15.38)	3 (4.62)	1 (1.54)
East Midlands	50	34 (68.00)	7 (14.00)	3 (6.00)	2 (4.00)
Unrecorded	40	29 (72.50)	8 (20.00)	1 (2.50)	0 (0.00)
West Midlands	35	26 (74.29)	3 (8.57)	2 (5.71)	1 (2.86)
South West	33	20 (60.61)	5 (15.15)	4 (12.12)	2 (6.06)
Scotland	23	16 (69.57)	3 (13.04)	2 (8.70)	1 (4.35)
North East	19	13 (68.42)	6 (31.58) *	0 (0.00)	0 (0.00)
Wales	14	9 (64.29)	0 (0.00)	3 (21.43) *	0 (0.00)
Northern Ireland	2	2 (100)	0 (0.00)	0 (0.00)	0 (0.00)

* Indicates a result that is significantly different (*p* < 0.05) to the rest of the UK.

**Table 5:** Descriptive and multivariable logistic regression results for risk factors associated with a severe grade of disease in dogs affected by heat-related illness (HRI) under primary veterinary care in the UK from 2016 to 2018.

Variable	Category	*n* (794)	Severe Cases (%)	Odds Ratio	95% Confidence Interval	*p*-Value
Age (years)						0.008
	<2	129	11 (8.53)	Reference		
	2 to <4	207	26 (12.56)	2.04	0.94–4.44	0.072
	4 to <6	132	22 (16.67)	3.12	1.37–7.08	0.007
	6 to <8	108	14 (12.96)	2.53	1.04–6.13	0.041
	8 to <10	70	9 (12.86)	2.57	0.95–6.96	0.064
	10 to <12	61	7 (11.48)	1.77	0.61–5.11	0.291
	12+	83	22 (26.51)	5.89	2.51–13.82	<0.001
	Unrecorded	4	0 (0.00)			
Bodyweight (kg)						<0.001
	<10	141	8 (5.67)	Reference		
	10 to <20	210	26 (12.38)	2.71	1.17–6.28	0.020
	20 to <30	141	19 (13.48)	2.41	0.99–5.87	0.052
	30 to <40	80	9 (11.25)	2.18	0.79–6.03	0.134
	40 to <50	24	4 (16.67)	3.62	0.97–13.57	0.056
	50+	10	2 (20.00)	4.30	0.76–24.4	0.100
	Unrecorded	188	43 (22.87)	6.46	2.84–14.71	<0.001
Trigger						0.010
	Exertion	411	49 (11.92)	Reference		
	Blankets	2	1 (50.00)	5.64	0.32–99.7	0.238
	Building	18	5 (27.78)	2.21	0.68–7.25	0.189
	Environmental	81	4 (4.94)	0.37	0.13–1.08	0.069
	Treatment	27	3 (11.11)	1.15	0.32–4.12	0.825
	Unrecorded	220	40 (18.18)	1.69	1.05–2.72	0.031
	Vehicular	35	9 (25.71)	3.03	1.28–7.15	0.011

**Table 6:** Descriptive and multivariable logistic regression results for risk factors associated with a fatal outcome in dogs affected by heat-related illness (HRI) under primary veterinary care in the UK from 2016 to 2018.

Variable	Category	*n* = 856	Severe Cases (%)	Odds Ratio	95% Confidence Interval	*p*-Value
Age (years)						<0.001
	<2	139	7 (5.04)	Reference		
	2 to <4	224	18 (8.04)	1.47	0.52–4.13	0.469
	4 to <6	144	16 (11.11)	1.66	0.56–4.90	0.358
	6 to <8	119	14 (11.76)	2.98	0.99–8.92	0.051
	8 to <10	76	9 (11.84)	3.55	1.03–12.22	0.044
	10 to <12	65	11 (16.92)	7.29	2.15–24.74	0.001
	12+	84	23 (27.38)	8.87	2.87–27.41	<0.001
	Unrecorded	5	0 (0.00)			
Skull shape						0.008
	Mesocephalic	377	37 (9.81)	Reference		
	Dolichocephalic	66	9 (13.64)	1.06	0.39–2.90	0.908
	Brachycephalic cross	6	0 (0.00)			
	Brachycephalic	287	42 (14.63)	3.01	1.60–5.67	<0.001
	Not applicable	120	10 (8.33)	0.86	0.35–2.15	0.748
VetCompass Clinical Grade						<0.001
	Mild	317	7 (2.21)	Reference		
	Moderate	366	20 (5.46)	2.70	1.11–6.55	0.029
	Severe	111	63 (56.76)	64.92	27.12–155.43	<0.001
	Unclassified	62	8 (12.90)	8.05	2.73–23.74	<0.001

## Data Availability

Supporting data are available via this link: https://rvc-repository.worktribe.com/output/1558421.
